# Breaking down the costs for breast cancer: Insights from Sweden's national quality register

**DOI:** 10.1016/j.breast.2025.104646

**Published:** 2025-11-11

**Authors:** Xiaoyang Du, Leo Gkekos, Balram Rai, Anna L.V. Johansson, Irma Fredriksson, Mattias Rantalainen, Emelie Heintz, Shuang Hao, Mark Clements

**Affiliations:** a Department of Medical Epidemiology and Biostatistics, Karolinska Institutet, Stockholm, Sweden; b Cancer Registry of Norway, Norwegian Institute of Public Health, Oslo, Norway; c Department of Molecular Medicine and Surgery, Karolinska Institutet, Stockholm, Sweden; d Department of Breast, Endocrine Tumors and Sarcoma, Karolinska Cancer Comprehensive Center, Karolinska University Hospital, Stockholm, Sweden; e Department of Learning, Informatics, Management and Ethics, Karolinska Institutet, Stockholm, Sweden; f Stockholm Center for Health Economics, Stockholm Health Care Services, Region Stockholm, Sweden

**Keywords:** Breast cancer, Health economics, Cost-of-illness, National register, Sweden

## Abstract

**Background:**

With new emerging technologies for diagnostics and treatment for breast cancer, there is a demand for updated breast cancer costs based on current clinical practice. The objectives of this study were to estimate recent societal costs of breast cancer in Sweden and provide population-based patient-level cost estimates for health economic evaluations.

**Methods:**

This prevalence-based cost-of-illness study was based on 2019 data linking multiple Swedish national registers. The analysis employed a societal perspective considering direct health care, informal care, and productivity losses. Total costs were estimated using a bottom-up micro-costing approach. Direct costs per patient-year were also estimated by subgroups, including age group, breast cancer subtype, breast cancer stage at diagnosis, and disease state defined by metastatic status.

**Findings:**

82,960 breast cancer patients diagnosed since 2008 were alive by the end of 2019. The annual societal cost of breast cancer in Sweden was €632 million, where the direct health care, informal care, and productivity losses accounted for 37 %, 5 %, and 57 %, respectively. The cost per capita was €61. Costs of direct health care, including inpatient/outpatient care and prescribed drugs, varied by subgroups, where younger age, higher stage, and more adverse subtypes were associated with higher costs per patient-year. Patients with a diagnosis of de novo metastatic cancer incurred the highest mean cost per patient-year.

**Conclusion:**

Breast cancer represents a large economic burden in Sweden. The mean cost estimates per patient-year are informative to future health economic evaluations for breast cancer screening and treatment.

## Highlights

• The estimated annual societal cost of breast cancer was €632 million in Sweden.
• Higher costs were observed in younger age, higher stages and more adverse subtypes.
• The highest cost incurred following a diagnosis of de novo metastatic cancer.
• First study to report stage-specific breast cancer costs in Sweden.
• Estimated cost per patient-year by subgroup can inform future economic evaluations.

## 1 Introduction

Breast cancer is the most diagnosed cancer and comprised 13.8 % of all cancer cases among European Union countries [1]. In Sweden, it is the most common cancer for women, with a 10-year prevalence of 63,017 cases in 2022 [2]. While its incidence stabilised over 15 years after a surge between the 1960s and 2000s, the mortality has declined [2]. Early detection through mammography screening might have significantly contributed to the reduction.

National breast cancer screening in Sweden was introduced in the 1980s. Since 2016, mammographic screening has been free of charge for women aged 40–74 years. Several Swedish studies assessing the effectiveness of screening at both regional and national level found that increased screening was associated with reductions in the incidence of advanced cancer and of breast cancer mortality [3,4]. For metastatic disease, advanced treatment through innovative targeted drugs may have prolonged survival, although potentially increasing health care expenditure [5,6].

The economic burden of breast cancer in Sweden has been investigated by two previous studies. Lidgren and colleagues estimated the societal costs of prevalent breast cancer cases diagnosed from 1958 to 2002 [7]. By using a top-down method, the estimated annual cost was 3 billion Swedish Kronor (SEK), largely due to productivity losses. Using aggregate data, Luengo-Fernandez and colleagues estimated that breast cancer cost €280 million for Sweden in 2009 [8]. With new emerging technologies for diagnostics and treatment, an updated estimation based on latest clinical practice is imperative. In addition, previous Swedish studies on breast cancer costs by different subgroups, including subtype and disease state, were conducted on small sample sizes, which findings may not be generalisable to a broader population [9,10]. By using the latest available register data, our study aimed to value breast cancer in Sweden to reflect the most recent breast cancer management, and estimate the patient-level costs to inform future health economic evaluations targeting breast cancer.

## 2 Method

This register- and prevalence-based study applied a societal perspective including direct and indirect costs. Individual-level resource utilisation in 2019 was extracted from the Breast Cancer Database Sweden (BCBaSe) 3.0 [11]. Resource use not included in BCBaSe 3.0 was estimated based on existing research (Fig. S1). Unit costs from year 2023 were directly applied; otherwise, adjusted to year 2023 using the consumer price index. Total costs were calculated using a bottom-up method multiplying the quantities of resources by the unit costs and converted to euro (€1 = 11.48 SEK, 2023 rate) [12]. Patient-level costs comprising inpatient/outpatient care and prescribed drugs were estimated by subgroups, including age group, subtype, stage at diagnosis, and disease state. Analyses were performed using R (version 4.2.3, R Foundation for Statistical Computing, Vienna, Austria) and Stata/BE 17.0 (Corp, College Station, Texas, USA).

### 2.1 Study population base

The study included women invited for a mammography screening in 2019 and individuals diagnosed with invasive or non-invasive breast cancer (C50 & D05, The International Classification of Diseases, 10th version, ICD-10) prior to December 31, 2019 in Sweden. The diagnosed individuals were retrieved through BCBaSe 3.0 (Supplementary A1) which has national coverage for breast cancer cases diagnosed from 2008. To extrapolate the costs to all prevalent cases in Sweden, we used cancer registrations from the NORDCAN database and the National Cancer Register to represent the cases diagnosed before 2008 (Fig. S1) [2].

### 2.2 Resources utilisation and unit costs

#### 2.2.1 Screening

Women aged between 40 and 74 years in Sweden are invited for a mammography screening every 18–24 month. A 78 % participation was reported in 2019–2020 by National Board of Health and Welfare [13]. We assumed that half of the screening population were screened in 2019 and 3 % of those participated were recalled for further investigation [14,15]. After clinical mammography, breast and axillary ultrasound, 33 % would have an ultrasound-guided core biopsy and/or fine needle aspiration. Procedure-specific prices were informed by the Swedish Institute for Health Economics (IHE) [14].

#### 2.2.2 Primary care

We identified clinically-detected cases outside screening in 2019 from BCBaSe 3.0 and assumed that the patients had one visit to primary care prior to a diagnosis of breast cancer. The primary care visit unit cost was extracted from the Swedish Association of Local Authorities and Regions (SKR) [16].

#### 2.2.3 Inpatient and outpatient care

Inpatient and outpatient care episodes were identified using the Nordic Diagnostic Related Groups (DRG, Table S3). The DRG unit costs were extracted from SKR [16]. To estimate the cost of pre-diagnostic procedures for clinically-detected cases, we included relevant DRG codes prior to a diagnosis of breast cancer (Supplementary A6).

#### 2.2.4 Pharmaceuticals

Thirty-six substances with indications for breast cancer were identified on the Swedish market by 2019 (Table S5). The quantities of drug prescriptions were extracted from BCBaSe 3.0. Unit costs of drugs were identified through The Dental and Pharmaceutical Benefits Agency database or from pharmacy websites (Supplementary A7). Costs of hospital-based requisition drugs were included in the inpatient and outpatient episodes through the DRG calculations [17].

#### 2.2.5 Palliative care

We identified breast cancer deaths in 2019 from the NORDCAN database and assumed that all deceased patients received palliative care, of which 10.5 % resided in nursing homes based on a Swedish register study [18]. Mean costs for palliative care per cancer patient were retrieved from a recent study in the Stockholm region [19].

#### 2.2.6 Informal care

Resource use of informal care was estimated using WAVE8 data from the Survey of Health, Ageing and Retirement in Europe (SHARE) project [20]. We used logistic regression to model the probability that a patient received informal care from a working-age (<65) caregiver for patients who were severely limited in daily activities and patients with terminal illness. The number of hours received from a working-age caregiver was modelled using linear regression. Informal care was valued at €28.5 per hour based on the average salary (including social security fees) in Sweden in 2023 (Supplementary A8) [21].

#### 2.2.7 Productivity losses

Productivity losses were estimated using the human capital method. For productivity losses due to morbidity, we identified the patients who had received long-term sick leave benefits and disability pension due to a breast cancer diagnosis as the main cause from BCBaSe 3.0. Employer-paid short-term sick leave up to 14 days was not recorded by any registers. The number of net days of individuals receiving long-term sick leave benefits and disability pensions were recorded in Swedish Social Insurance Agency and extracted from BCBaSe 3.0. Patients who had long-term sick leave were assumed to have had a 14-day sick leave beforehand. We valued the lost productivity until 65 years and applied a mean cost of €228 per day, based on the calculated average gross earning including social security fee in 2023 [21].

Breast cancer deaths in 2019 were identified in the National Cancer Register to analyse productivity losses due to premature mortality. The accumulated losses of years up to age 65 years were calculated using the background mortality from the Swedish general population together with a 3 % discount rate for future productivity losses. See detailed method from Hao et al. [22].

### 2.3 Subgroup analysis

Disease states were defined as: a primary diagnosis without distant metastases; primary diagnosis with de novo metastasis; and distant recurrence after primary diagnosis without metastasis. Multiple imputation was performed for missing M stage at diagnosis and for missing date of distant recurrence for breast cancer death cases (Supplementary A4 and Table S9).

Subtypes were defined using estrogen receptor (ER) status, progesterone receptor (PR) status, human epidermal growth factor receptor 2 (HER2) status, and Nottingham Histologic Grade (Table S1) [23]. We restricted to the subtypes of the first diagnosis for patients with multiple diagnoses. Multiple imputation was performed for missing subtype using the imputed datasets for M stage (Supplementary A4 and Table S9).

Stages were defined using the TNM classification 8th edition (Table S2) [24]. We restricted to the TNM status of the first diagnosis for patients with multiple diagnoses.

For the analysis of mean costs by different subgroups, we undertook a period analysis for 2019 with possible left truncation and right censoring for time since diagnosis, and for disease state, additionally split time since distant recurrence (Fig. S2). The average costs were calculated as the accumulated costs since diagnosis (and since distant recurrence for disease state) divided by the accumulated patient-years in 2019.

### 2.4 Extrapolation to the prevalent cases in Sweden

We extrapolated the costs of inpatient/outpatient care and prescribed drugs to include prevalent breast cancer cases diagnosed in Sweden before 2008. We performed the same period analysis for time since diagnosis using data from the National Cancer Register and calculated the accumulated patient-years by age group and by year since diagnosis to 2019. Average costs by patient-year were based on BCBaSe 3.0 data.

### 2.5 Sensitivity analysis

Sensitivity analyses were performed for the following: (a) varying proportion (40 % and 60 %) of screened women in 2019; (b) using resource use for inpatient/outpatient care in the KPP (cost-per-patient) system from SKR; and (c) using the proxy good method to estimate costs for informal care, which valued the care at the market price regardless of the working age of caregivers. An hourly cost of €20.7 was applied based on the average gross earning including social security fee of a personal care worker in health services in 2023 [21].

## 3 Results

### 3.1 Study population

In BCBaSe 3.0, there were 82,960 patients living with a diagnosis of breast cancer in Sweden by the end of 2019 (Table 1). Among 8734 incident cases in 2019, 4291 (49 %) were detected through screening. Of the 952 patients who died from breast cancer, approximately 60 % were aged ≥70.

**Table 1:** Description of the BCBaSe 3.0 study population (Sweden 2019).

Age group	Prevalent cases N (%)	Incident cases N (%)	All-cause deaths N (%)	Breast cancer deaths N (%)
<40	3308 (4)	335 (4)	20 (1)	18 (2)
40–49	13,144 (16)	1215 (14)	83 (3)	72 (8)
50–59	17,944 (22)	1618 (19)	177 (7)	125 (13)
60–69	26,063 (31)	2258 (26)	335 (13)	173 (18)
≥70	22,501 (27)	3308 (38)	1985 (76)	564 (59)
Total	82,960 (100)	8734 (100)	2600 (100)	952 (100)

### 3.2 Screening

We calculated that 705,913 women underwent mammography screening during 2019, of whom 21,177 were referred to a clinical work-up and 7059 underwent a biopsy. The estimated total cost was €63 million (Table 2).

**Table 2:** Costs of breast cancer by type of care in Sweden (2023 price, euro).

Type of care	Cost (€)	Percentage of total cost (%)
**Direct cost**	**281,905,564**	**37.41 %**
Screening	62,663,694	9.92 %
Primary care	572,965	0.09 %
Inpatient care	39,259,457	6.21 %
Outpatient care	72,841,119	11.53 %
Pharmaceuticals	20,588,297	3.26 %
Palliative care	40,393,556	6.39 %
**Informal care**	**34,251,936**	**5.42 %**
Severely limited in daily activities	29,763,136	4.72 %
Terminal illness	4,488,800	0.71 %
**Productivity loss**	**361,118,419**	**57.17 %**
Morbidity- Sick leave	184,679,943	29.24 %
Morbidity- Disability pension	8,103,690	1.28 %
Premature mortality	168,334,786	26.65 %
**Total cost**	**631,689,443**	**100 %**

### 3.3 Primary care

In BCBaSe 3.0, 4443 cases were detected outside screening in 2019. Assuming one primary care visit per case, the estimated primary care cost was €0.6 million.

### 3.4 Inpatient and outpatient care

In 2019, 4618 patients admitted to inpatient care for 5349 episodes. The most frequent DRG code – total mastectomy for malignant tumour – represented 36 % of inpatient care costs (Table S7). Outpatient care involved 16,762 patients with 91,456 episodes. The most frequent (52 %) DRG code was doctor visits for malignant breast diseases (Table S7). Pre-diagnosis procedures for clinically-detected cases contributed €0.2 million. Including cases before 2008, inpatient and outpatient care costs were estimated to be €39 million and €73 million, respectively (Table 2).

### 3.5 Prescribed drugs

In 2019, 36,218 patients were prescribed 26 substances for breast cancer, with tamoxifen, letrozole and anastrozole being the most frequent (Table S7). CDK4/6 inhibitors had the highest total cost at €6.4 million, accounting for 46 % of the total prescribed drugs costs (Table S8). Including cases before 2008, the estimated cost of prescribed pharmaceuticals was €21 million.

### 3.6 Palliative care

Among the 1365 deceased cases who received palliative care in 2019, 144 were nursing-home residents. The total cost of palliative care was estimated to be €40 million (Table 2).

### 3.7 Informal care

We included 74,352 patients that were diagnosed before 2019 and were alive in 2019 as the study base for receiving informal care due to their limitation in daily activities and assumed that 1.1 h and 1 h per day care were provided from inside and outside the household respectively. For terminal illness, an average number of 4.5 h of informal care per day were provided to 952 patients who died in 2019. The total cost of informal care was estimated to be €34.5 million (Table 2).

### 3.8 Productivity losses

The cost of productivity losses due to morbidity were estimated to be €193 million (Table 2), of which sick leave accounted for 96 %. An average of 146 net days were identified for 5690 patients (Table S7). For the 145 patients who received disability pension, the cost of productivity losses was €8 million. There were 352 patients aged <65 who died from breast cancer in 2019. Productivity losses due to premature mortality were estimated to be €168 million (Table 2).

### 3.9 Costs by subgroup

In the first year following a primary diagnosis, the highest costs per patient-year were observed for patients aged <40. The cost per patient-year decreased with increasing age. For costs per patient-year by cancer subtype, triple negative cancer costs were the highest, followed by luminal HER2+ cancers. For staging, stage III cancers incurred the highest cost (€14,974 per patient-year) in the first year following a diagnosis; for subsequent years, patients at stage IV had the highest mean cost per patient-year at €6,078, while the mean costs for stage I-III patients dropped below €1100. The highest mean cost per patient-year was observed following a diagnosis of de novo metastatic cancer (Table 3 and Fig. S3).

**Table 3:** Direct Costs combining inpatient/outpatient care and prescribed drugs, by subgroups (2023 price, euro).

Subgroup	First year of follow-up			Second and subsequent years of follow-up			Total years of follow-up		
	Total cost (€)	Total patient-years	Cost/patient-year (€)	Total cost (€)	Total patient-years	Cost/patient-year (€)	Total cost (€)	Total patient-years	Cost/patient-year (€)
**Breast cancer subtype**									
Luminal A like	41,937,303	4378	9578	14,216,773	39,032	364	56,154,076	43,410	1294
Luminal B like	18,943,215	1692	11,194	8,049,657	13,622	591	26,992,872	15,315	1763
Luminal HER2+	10,220,103	789	12,955	3,388,789	6480	523	13,608,892	7269	1872
HER2+	5,710,746	462	12,362	1,412,916	3423	413	7,123,661	3885	1834
Triple negative	12,060,203	1023	11,784	2,831,003	6861	413	14,891,206	7885	1889
**Breast cancer stage**									
Stage 0	4,277,268	561	7624	978,539	5014	195	5,194,317	5575	941
Stage I	31,967,902	3506	9119	8,033,018	33,365	241	39,075,689	36,870	1085
Stage II	38,523,974	3255	11,837	11,456,346	24,217	473	47,674,412	27,472	1819
Stage III	10,034,559	670	14,974	5,722,829	5449	1050	13,904,666	6119	2575
Stage IV	2,605,054	191	13,620	3,420,449	563	6078	6,025,503	754	7991
**Age group**									
<40	4,692,820	283	16,555	1,246,168	999	1248	5,938,988	1282	4632
40–49	14,808,300	1102	13,441	3,794,796	5342	710	18,603,096	6444	2887
50–59	18,524,942	1603	11,556	6,191,493	13,193	469	24,716,435	14,796	1670
60–69	22,230,516	2125	10,460	6,963,166	17,390	400	29,193,682	19,515	1496
≥70	28,614,990	3231	8856	11,691,711	32,494	360	40,306,701	35,725	1128
**Disease state**									
Primary diagnosis M0	85,806,509	8122	10,565	19,250,593	67,802	284	105,057,103	75,924	1384
De novo metastatic M1	2,605,054	191	13,620	3,420,449	563	6078	6,025,503	754	7991
Distant recurrence	3,566,038	363	9820	4,110,262	721	5697	7,676,298	1085	7078

### 3.10 Sensitivity analysis

Across the different sensitivity analyses, the total costs varied by −2 %–1.1 % (Fig. S4).

## 4 Discussion

Our study quantified the economic burden of breast cancer using individual-level data in 2019 from BCBaSe 3.0 and extrapolated costs to all prevalent cases in Sweden. The estimated annual societal cost was €632 million. The cost (excluding screening) per patient was €6852 and the cost per capita was €61.

The estimated total costs exceed earlier estimates, partly due to inflation and price changes, but also due to methodological differences. Firstly, unlike the 2002 study, we included palliative and informal care. Using the human capital method, indirect costs constituted 70 % of the total costs in the 2002 study [7], compared to approximately 57 % in this study. This reduction may be attributed to the decreased costs of premature mortality, which declined from 37 % in 2002 to 27 % in the present study. Such a decline may be explained by improved treatment, which is expected to increase survival [25]. Secondly, our bottom-up costing approach using individual-level data provides more precise estimates and allows costs by subgroup, unlike previous studies using top-down method or aggregated data [26,27]. The individual-level DRG episodes extracted from BCBaSe 3.0, in contrast to using the aggregate cost from KPP [16], allowed us to apply broader patient inclusion criteria, for example, including patients with metastatic cancer originating from breast.

Another strength of our study is the reporting of patient-level cost estimates by various subgroups, which can be used as cost inputs for health economic evaluations. Higher costs were observed in younger age groups. Patients aged <40 at diagnosis presented a higher proportion of more adverse subtypes (Table S6), which may require multimodal and more intensive treatments with higher expected expenditure. Previous Swedish studies identified HER2+ disease as the most costly subtype [9], while triple negative cancers cost the most in the present study; although cost differences among subtypes in our study were small. In contrast, studies in other countries reported a surge in the costs of HER2+ cancer [28,29]. This discrepancy may be driven by differences in the care components and treatment durations. For example, the Canadian study included home care and palliative care and reported costs per case given stage and treatment durations. The largest cost increase for HER2+ cancers occurred in stage IV due to cost changes in systemic therapy, whereas the cost differences among stages in other subtypes were smaller [29]. Costs by stage have been widely studied in other settings, where costs are consistently higher for higher stages [30,31]. To the best of our knowledge, this is the first study to report stage-specific breast cancer costs in Sweden. The highest initial costs occurred at Stage III cancer following a primary diagnosis, whereas stage IV cancers incurred significantly higher costs than lower stages in the subsequent years, primarily due to prescribed drugs (Table S10).

Lidgren and colleagues investigated disease state-specific costs for breast cancer using a survey design [10]. Using register data, we observed a similar pattern, where costs dropped from the first to the second year. The highest costs were observed in the first year following a diagnosis of de novo metastatic cancer. Most health care events and higher total costs emanated from outpatient care following a diagnosis of non-metastatic cancer. Markedly higher costs in prescribed drug were seen in the metastatic settings (Table S10). Expensive targeted drugs as first-line treatment to metastatic disease, such as CDK 4/6 inhibitors, have been recommended in the Swedish national guidelines [23]. New targeted therapies (ADC inhibitors and PARP inhibitors, etc.) for breast cancer have been approved after 2019. With the increasing use of these agents, a continued increase in prescribed drug costs is expected. For disease states defined in the metastatic settings, the number of patients who had had events decreased over time, leading to imprecise estimates. Consequently, we truncated the follow-up for those disease states to 6 years (Fig. S3).

Some limitations should be noted. Firstly, resource use for screening, informal care and palliative care were estimated based on existing research. Screening attendance rates and the proportion of diagnosis (20 %) after a recall for clinical mammography were informed by national reports [4,15]. The estimate of diagnosed cases in the primary analysis was close to the number of screening-detected cases observed in BCBaSe 3.0 (4236 vs. 4291), indicating the robustness of our assumptions. However, procedure costs for benign cases and for molecular diagnostics through gene expression assays were not included, yielding underestimation of diagnostic costs. Secondly, as WAVE8 of the SHARE lacked reporting of the care hours per day for patients severely limited in daily activities, we used estimates from a previous study based on WAVE2 [22]. The care pattern may have changed over years. In addition, our calculations for informal care did not included patients with primary diagnoses before 2008. Moreover, we did not link the Palliative Care Register due to incomplete coverage (>50 %). The unit costs for palliative care were extracted from an end-of-life study on cancer patients in Stockholm Region; these costs are not breast cancer-specific and may be less representative for other regions in Sweden [19]. Thirdly, pharmaceutical costs may be undervalued because outpatient DRGs for intravascular drug delivery were not fully captured by BCBaSe 3.0. Fourthly, productivity losses were measured using the human capital method, which will lead to higher estimates compared with methods that consider work replacement. Lastly, primary care accounted for 0.1 % of the total breast cancer costs, driven by resource use related to symptomatic detection. This was lower than the 0.9 % reported by IHE in year 2013 [32]. However, in most cases breast cancer is treated in specialist care in Sweden; the potential underestimation of cost for post-diagnosis primary care is likely minor.

Despite some cost underestimation, the total estimated cost of breast cancer is higher than that of other cancer types in Sweden, given a higher prevalence [22,33,34]. In Europe, breast cancer ranks second in total cancer costs, exceeded only by lung cancer [8]. Our study underscores that breast cancer is a significant public health concern and a substantial economic burden for Sweden. From a policy perspective, adopting more cost-effective screening strategies and treatment modalities could enhance economic sustainability by enabling earlier breast cancer detection and improved survival outcomes.

## 5 Conclusion

Breast cancer constitutes a massive annual economic burden at €632 million in Sweden. By studying the patient-level costs by subgroups, we are able to provide cost estimates for future health economic evaluations targeting breast cancer.

## CRediT authorship contribution statement

**Xiaoyang Du:** Writing – review & editing, Writing – original draft, Visualization, Methodology, Investigation, Formal analysis, Conceptualization. **Leo Gkekos:** Writing – review & editing, Investigation. **Balram Rai:** Writing – review & editing, Investigation. **Anna L.V. Johansson:** Writing – review & editing, Investigation. **Irma Fredriksson:** Writing – review & editing, Investigation, Data curation. **Mattias Rantalainen:** Writing – review & editing, Supervision, Funding acquisition. **Emelie Heintz:** Writing – review & editing, Supervision, Methodology. **Shuang Hao:** Writing – review & editing, Supervision, Methodology, Conceptualization. **Mark Clements:** Writing – review & editing, Writing – original draft, Supervision, Resources, Methodology, Investigation, Funding acquisition, Formal analysis, Conceptualization.

## Ethics

The study was conducted under the ethical permits Dnr 2019–02610 and Dnr 2020-00886 from the Swedish Ethical Review Authority.

## Declaration of competing interest

The authors declare the following financial interests/personal relationships which may be considered as potential competing interests: Mark Clements reports financial support was provided by Cancerfonden. Mark Clements reports financial support was provided by Vetenskapsrådet. Mark Clements reports financial support was provided by European Commission. Mattias Rantalainen reports financial support was provided by Sweden's Innovation Agency. Mattias Rantalainen reports a relationship with Stratipath AB that includes: equity or stocks. If there are other authors, they declare that they have no known competing financial interests or personal relationships that could have appeared to influence the work reported in this paper.
